# The Medical Treatment of the Late Duke of Wellington

**Published:** 1853-01

**Authors:** 


*The Medical Treatment of the late Duke of Wellington.*—The Duke of Wellington was supposed to be in his usual health until the morning of the day of his death; how far this opinion may be correct will subsequently appear. He had been engaged until dusk of the preceding evening reading in the report of the Oxford University Commission, and did not suspend his labors until compelled by inability to distinguish the print; having at the time noticed the light on the opposite coast, he observed that it was the "darkness," and not the failure of his sight, which caused the print to "bother" him. He dined heartily shortly afterwards, at seven o'clock, and took for dinner *mock turtle-soup, turbot, venison and pudding.* As was his usual practice, he drank neither wine nor spirit. He retired to bed before ten o'clock, and during the night visited the closet. The appearances found there showed that whilst the functions of the bowels were healthily performed, the Duke, contrary to his habit, must have returned hastily to bed, probably in pain. His Grace's valet, Mr. Kendal, who called him at his usual hour, shortly after six o'clock, observed that his master was not well, and that his breathing seemed oppressed. His Grace not appearing disposed to get up, his attendant, after remaining sometime in the room, left him until half past seven o'clock. Returning at this time he was directed by the Duke to send for "the apothecary." This he (Mr. Kendall) immediately did, and Mr. Hulke, of Deal was in attendance about nine. He found his Grace complaining much of *pain across the chest and at the pit of the stomach. His tongue was furred; he had distressing eructations and his pulse was irregular.* It was intimated by Mr. Hulke, that he would send a draught, and he recommended that his grace in the meantime should take a little warm tea and toast. Mr. Kendal endeavored shortly afterwards to act on this recommendation, but the Duke seemed unable to swallow the tea. He became sick and threw up a portion of the *venison* which he had taken the evening before. This piece of meat had not been altered in appearance by the process of digestion. A general convulsive attack ensued, of some minutes duration. After the fit the Duke to some extent recovered his consciousness. He laid on his back, his favorite position when in bed, with his hands clasped, and placed at the back of his head, his eyes occasionally following persons in the room. Mr. Hulke was immediately sent for again, and speedily returned, accompanied by Dr. M'Arthur. His Grace had another, but less severe convulsive attack, between eleven and twelve o'clock. Further medical assistance was sought for from London, and telegraphic messages were sent to Dr. Hume, who had long been the medical attendant of the Duke, to Dr. Robert Ferguson a friend of Dr. Hume, and to Dr. Williams, who alone of the three happened to be in town, but who, unfortunately did not arrive until the Duke had for several hours ceased to live. This lamentable event occurred a few minutes before half-past three o'clock.

After the first convulsive attack, the Duke's exhaustion rapidly increased, and his breathing became much embarrassed; he had slight twitchings in one arm, but no paralysis. When an effort was made to give him either medicine or drink, his Grace generally exhibited reluctance to take it, pushed away whatever was offered to him, and showed his usual dislike to be interfered with. The treatment employed consisted of a mustard poultice applied to the pit of the stomach by the valet; a mustard emetic, partially administered, and without action; a dose of calomel and small quantities of stimulants were offered to the patient, but were not swallowed. For some time before his death, his Grace had been removed to a chair, to relieve the difficulty of breathing; but the medical attendants finding that his pulse, already extremely feeble, became in that position, still weaker, his Grace was again restored to the recumbent posture.

Such then appears to have been the progress of the short and fatal illness of the great Duke of Wellington—

an illness that terminated so calmly, that not an expiring movement was observable by the medical attendants.

Let us seek, from this history, to discover the cause of the terrible calamity the nation so deeply laments.

It must, in the first place, be remembered, that the atmosphere of the sea-coast, when at all cold, causes, in the aged, or in debilitated persons, more or less accumulation of blood in the internal organs, and more or less consequent impairment and irregularity of their functions. During some days preceding the 14th of September, the day of the Duke's death, there had been a hot midday sun, a considerable wind, chiefly from the north, and the evenings and nights were cold and chilly. The thermometer, on the night preceding the fatal event, was only six degrees above the freezing point; on the preceding day it had been up to 92°. No precautions were taken to obviate the effects of such a change on the aged and necessarily weak system of the Duke; and the pallor of his countenance observed on the preceding Sunday, showed that this influence was telling on the circulation. The stomach was thus ill prepared to receive a hearty dinner; and the difficulties of that organ were further increased by receiving a considerable quantity of food imperfectly masticated, in consequence of the Duke's loss of teeth. Nor was the process of digestion promoted, or the powers of the stomach and heart invigorated, by the use of stimuli. The stomach therefore contained a mass of undigested food, and became distended with flatus; the functions of the lungs were impeded; the heart's action was disturbed; the nervous system participated in the morbid processes going on; and as a child would have convulsions under similar circumstances, so had the Duke of Wellington, who becoming exhausted by the disturbed and enfeebled condition of his nervous and circulating systems, rapidly sunk and expired. Why or wherefore such an attack should be called "epilepsy," we are at a loss to conceive. The certificate of death, at least as it has appeared in the journals, is not correctly expressed.—*London Lancet.*

